# LEMABE: a novel framework to improve analogy-based software cost estimation using learnable evolution model

**DOI:** 10.7717/peerj-cs.800

**Published:** 2022-01-03

**Authors:** Maedeh Dashti, Taghi Javdani Gandomani, Dariush Hasanpoor Adeh, Hazura Zulzalil, Abu Bakar Md Sultan

**Affiliations:** 1 Data Science Research Center, Shahrekord University, Shahrekord, Chaharmahal and Bakhtiari, Iran; 2 Department of Computer Science, Shahrekord University, Shahrekord, Chaharmahal and Bakhtiari, Iran; 3 Department of Electrical and Computer Engineering, Isfahan University of Technology, Isfahan, Isfahan, Iran; 4 Department of Software Engineering and Information Systems, Universiti Putra Malaysia, Serdang, Selangor, Malaysia

**Keywords:** Software cost estimation, Learnable evolution model (LEM), Analogy-based estimation, Features weighting optimization, Software project management, Software cost estimation framework

## Abstract

One of the most important and critical factors in software projects is the proper cost estimation. This activity, which has to be done prior to the beginning of a project in the initial stage, always encounters several challenges and problems. However, due to the high significance and impact of the proper cost estimation, several approaches and methods have been proposed regarding how to perform cost estimation, in which the analogy-based approach is one of the most popular ones. In recent years, many attempts have been made to employ suitable techniques and methods in this approach in order to improve estimation accuracy. However, achieving improved estimation accuracy in these techniques is still an appropriate research topic. To improve software development cost estimation, the current study has investigated the effect of the LEM algorithm on optimization of features weighting and proposed a new method as well. In this research, the effectiveness of this algorithm has been examined on two datasets, Desharnais and Maxwell. Then, MMRE, PRED (0.25), and MdMRE criteria have been used to evaluate and compare the proposed method against other evolutionary algorithms. Employing the proposed method showed considerable improvement in estimating software cost estimation.

## Introduction

The process of software development is a set of software engineering activities designed and planned to manage the life cycle of a software product. Generally, a software product life cycle can be divided into three major phases, *i.e*., planning, development, and maintenance (Boehm, 1984). In the software development process, particular principles and rules have to be defined for each phase, among which cost estimation at the beginning of a project makes up a critical activity in the planning (Xu & Khoshgoftaar, 2004). Because of its impact on the execution of the project, cost estimation is also considered as one of the most important factors of success or failure of projects (Dillibabu & Krishnaiah, 2005).

In the past two decades, significant efforts have been made in order to estimate the required cost and time of a software project more accurately to do more effective scheduling, planning, and manage the product quality. Moreover, several techniques and models have been proposed mainly for simplifying the cost estimation process. Today, however, because of the substantial growth of the size and efficiency of software applications as well as the uncertainty of requirements, especially in Agile methodologies, achieving more accurate estimation is still a concern for many software experts (Keaveney & Conboy, 2006). Software project managers need a series of reliable techniques for proper estimation so that they can make the right decisions on managing the software product life cycle and delivering it to the market.

In the analogy-based estimation method, a comparative process based on the project’s features is performed. Even though the lack of providing accurate estimation due to the complexity levels of projects, as well as the difficulties in determining the relationship of project features, is the shortcoming of this method. Considering the complexity of this method, using machine learning methods and soft computing has been recommended in recent years for enhancing estimation accuracy (Li, Xie & Goh, 2009; Bardsiri et al., 2013; Idri, Hosni & Abran, 2016). These methods can be used either directly through the process of project selection or feature weighting (Tosun, Turhan & Bener, 2009) or applied indirectly through machine learning methods such as artificial neural networks or fuzzy neural networks (Kazemifard et al., 2011; Moosavi & Bardsiri, 2017).

One of the emerging algorithms in artificial intelligence is the learnable evolution model (LEM). This model is one of the non-Darwinian evolutionary computation algorithms using machine learning for guiding the evolutionary process. This model suggests a new type of operators for creating the initial population that can act two or more times quickly in terms of the number of evolutionary steps (Wojtusiak & Michalski, 2004). This algorithm can contain a wide range of applications (Cervone, Kaufman & Michalski, 2000; Domanski et al., 2004; Jourdan et al., 2005; Cobos, Estupiñán & Pérez, 2011). LEM algorithm is able to perform successfully in solving complex optimization problems in the real world. So, it this study, we tried to investigate the usage of this algorithm in analogy-based software cost estimation.

The rest of the paper is structured as follows. In “Analogy-based Estimation Method”, the analogy-based estimation method is briefly introduced. Then, related work will be discussed in “Related Work”. The LEM algorithm is presented in “Learnable Evolution Model”, followed by “LEMABE Framework”, which introduces the proposed method. “Experiment Design” will discuss evaluation criteria, datasets, and implementation details. The results and empirical experiments using the proposed model will be presented in “Experiment Results”. Finally, “Conclusions” concludes the paper.

### Analogy-based estimation method

The analogy-based estimation is a very simple, efficient, and practical method in its nature. In this method, no particular formula is used, and in order to estimate a new project, it has to be compared with similar projects which have completed in the past, known as historical datasets or repositories. Analogy-based estimation consists of four parts, including the historical dataset, similarity function, K-nearest neighbors, and solution function. Moreover, its process consists of the following steps:

1. Creating the historical dataset accessible through real or artificial datasets;
2. Obtaining features associated with a new project in a way that they are in line with historical datasets;
3. Using a predefined similarity function such as the Euclidean or Manhattan distance functions so that can be retrieved the projects similar to the new project;
4. The new project’s cost is estimated using the solution function.

In the following, each component of the analogy-based estimation system has been separately described briefly.

### Similarity function

The similarity function is the core of the analogy-based estimation method, in which the degree of similarity between two different projects is calculated. The general form of the similarity function is as follows:

(1) $$sim\left( {p.{p}^{\prime}} \right) = f\left( {Lsim\left( {{f_1}.f_1^{\prime}} \right). Lsim\left( {{f_2}.f_2^{\prime}} \right). \ldots Lsim\left( {{f_n}.f_n^{\prime}} \right)} \right)$$

where p and p′ are indicative of the new and old projects in the repository, ${f_i}$ and $f_i^{'}$ indicate the value of the i^th^ feature in the mentioned projects, n is the total number of features in each project, and $Lsim\left( . \right)$ function calculates the degree of similarity between two corresponding features of the projects. $Lsim\left( . \right)$ and $f\left( . \right)$ functions show the general structure of the similarity function. Among the various types of similarity functions, Euclidean distance similarity function (ES) and the Manhattan distance similarity function (MS) are the most useful ones in the software development cost estimation domain. In mathematics, the Euclidean distance is the ordinary distance between two points used in comparing the distances by optimization problems (Shepperd & Schofield, 1997). The similarity between the two projects is obtained using the Euclidean Similarity distance through Eq. (2) (Hong & Kim, 2000).

(2) $$sim\left( {p.{p}^{\prime}} \right) = \displaystyle{1 \over {\left[ {\sqrt {\mathop \sum_{i = 1}^n {w_i}Dis\left( {{f_i}.f_i^{\prime}} \right) + \delta } } \right]}}\quad \delta = 0.0001$$

$\left\{ \matrix{ \left( {f_i} - f_i^{'} \right)^2  if\; \; features\; are\; numeric \cr {\hskip-27pt}1  if\; \; features\; are\; numeric\; and\; {f_i} = f_i^{'} \cr {\hskip-27pt}0  if\; \; features\; are\; numeric\; and\; {f_i} \ne f_i^{'} $

where ${w_i}$ is the *i*^th^ feature’s weight, and its value is between 0 and 1. Also, $\delta = 0.0001$ is a small fixed number that is placed in the equation to prevent division by zero.

The Manhattan Similarity distance (MS), also known as the city block distance-based similarity, is a type of Euclidean distance in which the distance between two points is the sum of the absolute differences of their coordinates. It is shown in Eq. (3) (Gardner, 1997).

(3) $$sim\left( {p.{p}^{\prime}} \right) = \displaystyle{1 \over {\left[ {\mathop \sum_{i = 1}^n {w_i}Dis\left( {{f_i}.f_i^{\prime}} \right) + \delta } \right]}}\quad \delta = 0.0001$$

$\left\{ \matrix{ \left| {f_i} - f_i^{'} \right|  if\; \; features\; are\; numeric \cr {\hskip-27pt}1  if\; \; features\; are\; numeric\; and\; {f_i} = f_i^{'} \cr {\hskip-27pt}0  if\; \; features\; are\; numeric\; and\; {f_i} \ne f_i^{'}$

Since the dataset is developed base on trial and error, there is no obligation in choosing the Euclidean function or Manhattan function. The nature of the projects and the level of their normality in the data set can have a significant impact on the performance of similarity functions. In some previous works, several other similarity functions have been used, including rank mean similarity (Walkerden & Jeffery, 1997), maximum distance similarity, Minkowski similarity (Angelis & Stamelos, 2000), but there is no specific method to show the best similarity function (Li & Ruhe, 2008; Li, Xie & Goh, 2008). In a systematic literature review, Wen et al. (2012) identified 8 machine learning models for software effort estimation. Most of these studies have been done on the Euclidean, Manhattan, and Minkowski distance, and a limited number of them have determined the similarity degree between two projects using Grey distance (Azzeh, Neagu & Cowling, 2010).

### K-Nearest Neighbor

The KNN algorithm is an example of instance-based learning, in which the training datasets are stored, and then, classification for a record that has not been classified yet is done simply by comparing it with the most similar records in the training dataset (Larose & Larose, 2014). The number of KNN is known as a vital parameter very effective on accuracy. Regarding the selection of *k* value, if *k* is very small, the effect outliers or unusual observations (noise) increases, and if *k* value is very large, the local behavior is ignored. Some of the papers have suggestions for how to keep *k* value fixed (Chiu & Huang, 2007), but *k* value is dynamic in most papers.

### Solution function

In this section, it will be clarified how we can use similar projects and combine them in order to estimate costs for the new project. The following evaluation methods have been used as a basis for the solution function in this research:

- Closest analogy (Walkerden & Jeffery, 1999);
- Unweighted mean;
- Median;
- Inverse distance weighted mean.

Mean is known as a classic criterion, which has a central tendency. In addition, the average software cost (*k* values) can be calculated when *k* > 1. Median is another classic criterion with central tendency. In this criterion, software costs (*k* values) can be calculated when *k* > 2. In comparison with mean, the median is a stronger statistical criterion because it is more sensitive to outliers. At the same time, number of outliers will growth by increasing the number of projects. Inverse distance weighted mean shows that similar projects are more important than less similar ones. Equation (4) shows the formula of inverse distance weighted mean (Kadoda et al., 2000).

(4) $$\widehat {{C_p}} = \mathop \sum \limits_{k = 1}^n \displaystyle{{Sim\left( {P.{P_k}} \right)} \over {\mathop \sum _{i = 1}^n Sim\left( {P.{P_k}} \right)}}{C_{pk}}$$

where *P* shows the project whose cost has to be estimated. *P_k_* is the *k*^th^ similar project. $Sim\left( {P.{P_k}} \right)$ is indicative of the similarity between projects *P* and *P_k_*, and *C_pk_* is the cost of the most similar project to *P_k_*.

Because the solution function is one of the most influential components in estimating accuracy, several studies have attempted to improve its performance. Some studies have used only one solution function (Li & Ruhe, 2008), while other studies have considered several types of solution functions (Li, Xie & Goh, 2009).

## Related Work

A literature review shows that researchers have presented different classifications for software cost estimation methods based on underlying principles and factors. In a systematic review among 304 papers from 76 journals, Jorgensen and Shepperd identified 11 cost estimation methods and divided them into parametric and non-parametric models (Jorgensen & Shepperd, 2007). In parametric models, cost estimation is based on statistical and/or numerical analysis of historical datasets (Azzeh & Nassif, 2016). In contrast, in non-parametric models, it is based on optimization principles and artificial intelligence methods such as Artificial Neural Networks (ANN), Genetic Algorithm (GA), Analogy-based or Case-based Reasoning Estimation, Decision Tree, and so on.

The analogy-based estimation method is a case-based reasoning model (Kolodner, 2014), which was introduced by Sternberg (1977) for the first time. After that, in 1997, this method was used for improving cost estimation in software development effort estimation (Shepperd & Schofield, 1997). This method relies on previously completed projects to estimate the required development effort for the new ones.

Different methods have already been proposed for analogy-based estimation, and the focus of all of them has been on improving estimation accuracy. To do this, the correlation coefficient has been used to improve the ABE performance, which can be the feature selection or the feature weighting.

In the normal analogy-based method, each of the project’s features is independent and has similar rates of effectiveness. For more accurate estimation, Auer et al. (2006) suggested that each of the features should have different rates of efficiency. In this case, the features that have a weak correlation, *i.e*., less impact, are given less weight, and the features with stronger correlations are given higher weight, and features without correlation are removed. Some studies using Traditional methods (Mendes, Mosley & Counsell, 2003; Phannachitta et al., 2013) and machine learning methods (Benala & Bandarupalli, 2016; Zare, Zare & Fallahnezhad, 2016; Benala & Mall, 2018; Ezghari & Zahi, 2018) have demonstrated an improvement in ABE performance. The analogy-based estimation has been widely used to enhance the accuracy of software cost estimation. Using optimization algorithms, especially swarm intelligence, has been recommended by many researchers in order to improve the accuracy of software development estimation (Liu et al., 2014; Satapathy, Acharya & Rath, 2016).

Pawlak (2012) used a weighting technique called Rough set analysis, in which the appropriate weight for independent features was determined based on a series of predefined criteria, but most of the studies in this field have been carried out using soft computing and metaheuristic algorithms (Huang & Chiu, 2006; Wu, Li & Bao, 2018). For example, genetic algorithm (GA) is known to be one of the most popular methods for optimizing and weighing features, and even combining genetic algorithms with other techniques, including regression methods, has been used to the accuracy of cost estimates improve accuracy (Chiu & Huang, 2007; Huang & Chiu, 2006; Oliveira et al., 2010). In addition, fuzzy systems (Azzeh, Neagu & Cowling, 2008), evolutionary algorithms, and artificial neural networks (Li, Xie & Goh, 2009; Azzeh, 2012) have been used for ABE adjustment.

In 2015, Azzeh, Nassif & Minku (2015) evaluated comparative analogy-based methods and concluded that linear adjustment methods could produce better solutions. On the other hand, some researchers have proposed several feature selection methods, some of whom did that through information (Li, Xie & Goh, 2009) and some others through optimization algorithms (Wang et al., 2007).

### Learnable evolution model

All common methods of evolutionary computations have been inspired by Darwin’s theory’s principles: “…one general law, leading to the advancement of all organic beings, namely, multiply, vary, let the strongest life and the weakest die” (Darwin, 1987). Darwin’s evolutionary computations are semi-blind, in which operators such as mutation (an unusual reproduction with variety), crossover (sexual reconstruction or recombination), and selection (survival of the fittest) were used to produce new population. In this type of evolution, individuals of the new population are not guided by the trained individuals of the previous population, but there is a trial and error process that is done in a parallel way. The main idea of LEM comes from a combination of evolutionary search and machine learning methods introduced in 2000 (Michalski, 2000). This strategy runs a machine learning model so that it can identify those individuals performing better in doing tasks. These reasons are expressed as inductive hypotheses and then used to produce the new population of these individuals. In fact, the new population is produced using hypotheses related to individuals with high fitness in the previous population.

LEM may switch between the machine learning mode and Darwinian Evolution mode or rely completely on machine learning. If LEM only runs the machine learning mode, it is so repeatedly applied that machine learning reaches evolution for the new population. While working with both modes, LEM switches from one mode to another when it can meet the termination condition. The evolutionary process proceeds until the solution for LEM is satisfactory, or the allocated sources become exhausted (Wojtusiak & Michalski, 2006).

The evolution process in LEM begins with a number of individuals of one initial population. In evolutionary processing of individuals may demonstrate solutions to issue or instructions for generating Solutions. An overview of the LEM process is given below:

1. Creating population: the initial population is created randomly or based on a particular method
2. Running the machine learning mode
  - Extracting the extrema: two groups are selected from the present population members; one group with high performance, known as H-group in short, and one group with low performance, known as L-group in short. The value of these groups is obtained using the fitness function’s value.
  - Making hypotheses: one machine learning method for describing the H-group and L-group is applied so that it can distinguish the two groups. While describing H-group or L-group, the evolution history, *i.e*., the previous population or description of previous populations, has to be taken into account.
  - Creating a new population: to do so, the samples learned in the H-group have to be combined with the new population. Describing samples is done either randomly or in the form of rules from the described samples.
  - Proceeding to step (2-a) and repeating the machine learning mode until it reaches the termination condition. Once the termination condition is met in the machine learning mode, one of the following actions can be taken:
    1) Ending the evolution process
    2) Repeating the process from stage 1; this operation is called start-over.
    3) Proceeding to stage 3
3. Running the Darwinian evolution mode: in this section, one of the methods of Darwinian evolution is run, *i.e*., one mutation, crossover, and selection is applied for producing the new population. This operation is ongoing until the Darwinian evolution mode reached the termination condition.
4. Alternating the modes: in this mode, we proceed to stage two and then keep switching between stages 2 and 3 until the LEM termination condition is met. The best-obtained individual is the evolution result.

**Figure 1:**
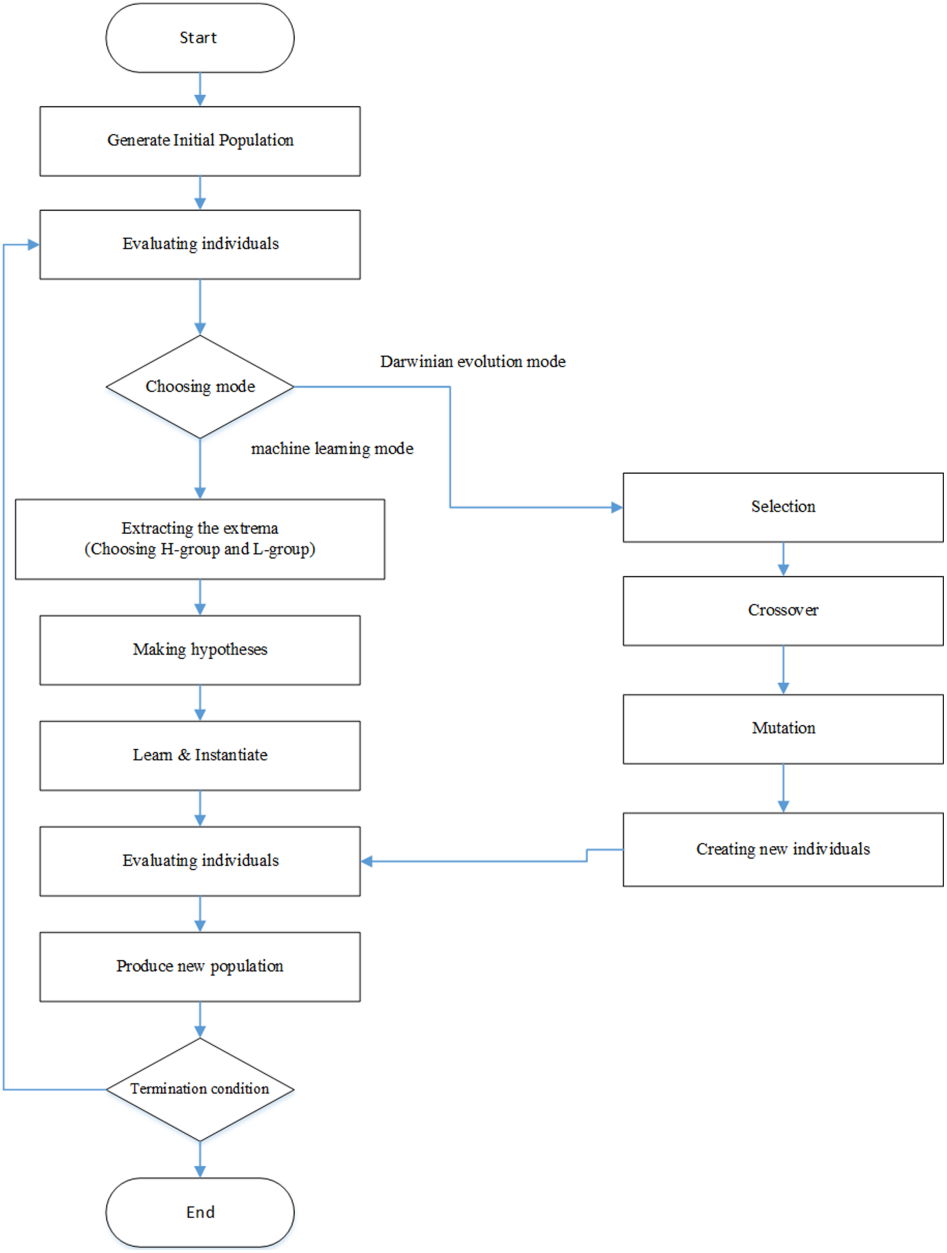
LEM algorithm flowchart.

The termination condition in the machine learning mode is fulfilled in a day if a particular level of performance is reached. In this stage, if the termination condition of LEM is not met yet, the start-over operation is run, or it proceeds to the termination condition of the Darwinian evolution mode. If LEM always selects the start-over operation, the evolution process will only be based on a repetitive program of the machine learning mode. This version of LEM operation is called uniLEM because it does not include the Darwinian evolution mode. If LEM uses both modes, it is called duoLEM. The flowchart of LEM algorithms has been illustrated in Fig. 1.

When LEM selects the start-over operation, it has to proceed to stage 1. One simple solution for running stage 1 is to describe a new population like in other evolutionary algorithms randomly. In LEM, however, this usually happens for the first time while running stage 1, and there are other methods such as select-elite, avoid-past-features, using suggestions, and creating another variant for running the start-over operation.

## Lemabe Framework

In ABE, a new project will be compared to completed projects. Therefore, it can be concluded that the accuracy of estimates is highly dependent on the correctness of comparisons. Due to the complex nature of software projects, determining the similarity between the two projects without considering the importance of each feature can have many negative effects. As mentioned earlier, in the ABE method, the comparison is made through a similarity function. Therefore, the proposed model emphasizes the improvement of the similarity function. In the proposed estimation model, the LEM algorithm is combined with the ABE method to increase the accuracy of the estimates. In fact, LEM is used specifically to find the most appropriate weight of features for use in the similarity function.

**Figure 2:**
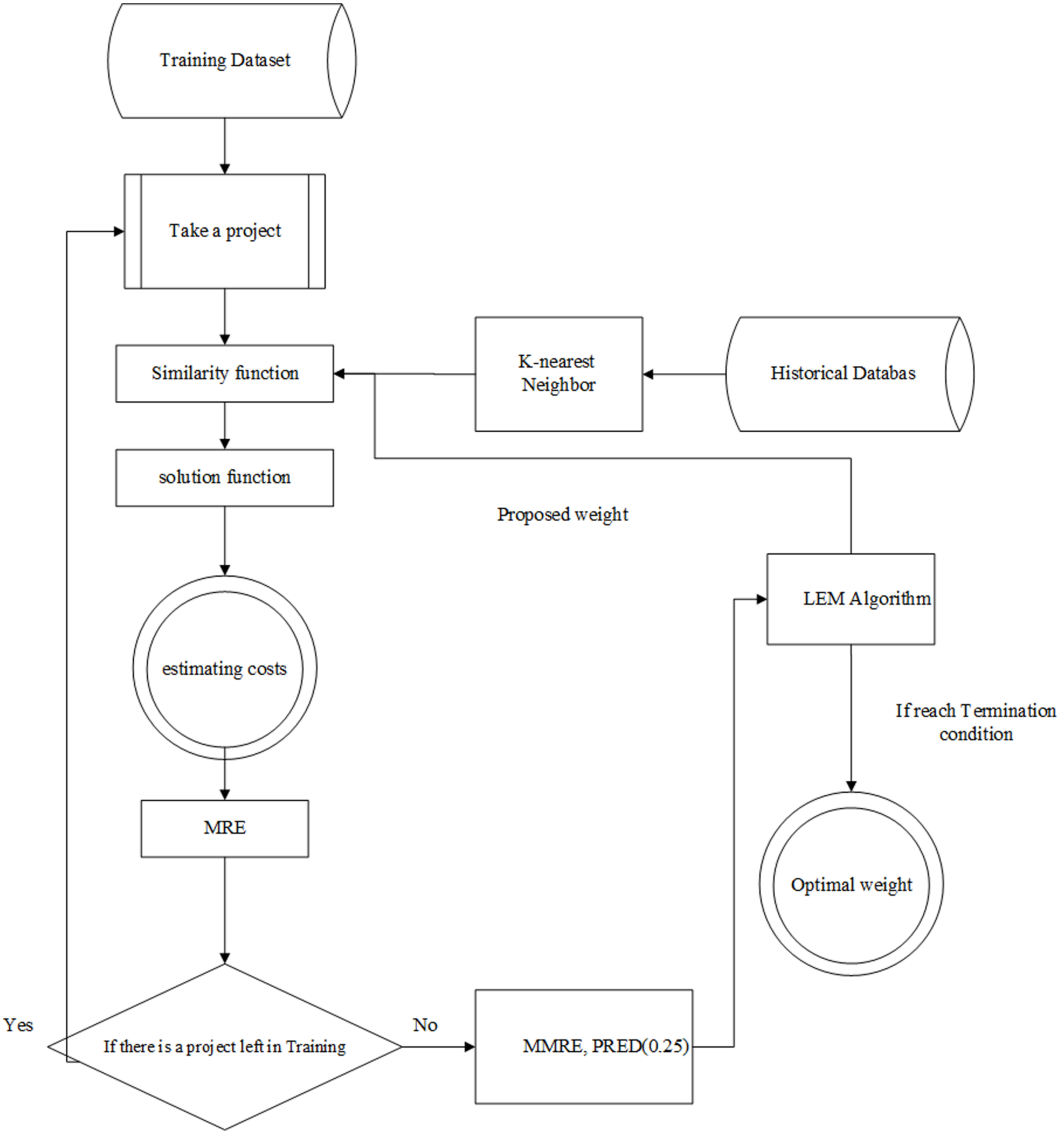
The architecture of training system.

**Figure 3:**
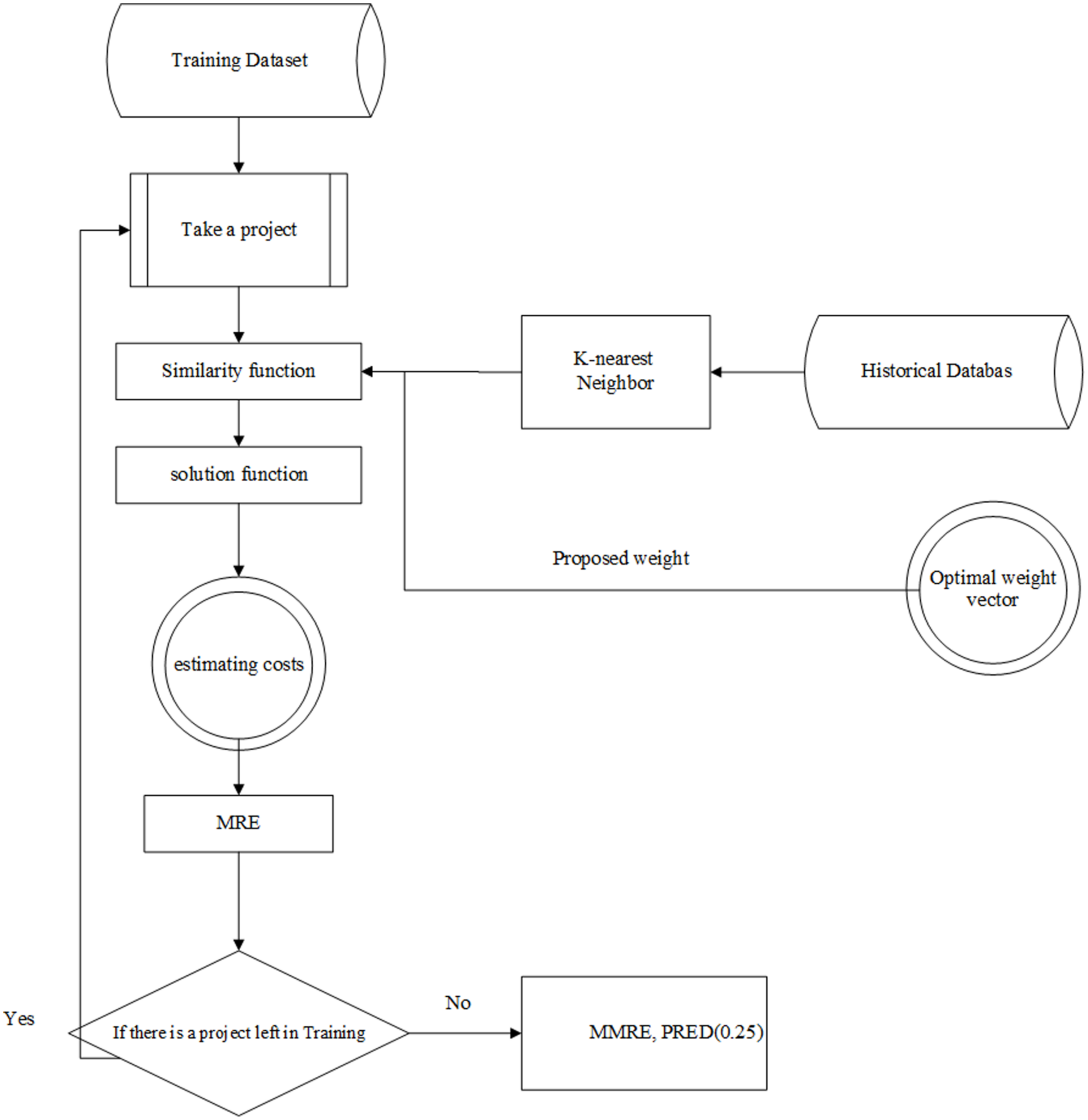
The architecture of the testing system.

The Learnable evolution model in analogy-based estimation (briefly LEMABE) consists of two phases, *i.e*., training and testing. The architecture of these phases is illustrated in Figs. 2 and 3, and the pseudo-code is shown in Table 1.

**Table 1:** Pseudo-code of LEMABE framework.

Algorithm LEMABE
I**nput: *f* (.) **Objective function
**Population Size**
**elite-ratio**
**Crossover Rate**
**Mutation rate**
*Step 1*: Initialize population POP
Cross over rate = 0.5
Mutation rate = 0.1
Set G = 0 (Generation number)
*Step 2*: From the training dataset, one project is selected as the new project to be estimated, while the others are treated as historical projects in the ABE system.
*Step 3*: In LEM’s parameter vector, encode the training project’s feature weights.
*Step 4*: Create a weight vector at random from the range [0, 1].
*Step 5*: Using a picked at random weight vector in pop, assess the similarity metric for the training project.
*Step 6*: Obtain the K closest analogies to the historical dataset
*Step 7*: Calculate solution function
*Step 8*: Repeat steps 2–7 until all of the training instances have been processed with the same random weight vector (produced for to begin with training instance).
*Step 9*: Calculate MRE for each individual using objective function. The objective function is (*MMRE* − *PRED* (0.25) + ∈). The ∈ is a minor positive constant that was purposely included to keep the denominator from being zero.
*Step 10*: To assess forecast accuracy, compute MMRE and PRED (0.25) and MdMRE.
*Step 11*: The Evolution Step // As long as the stopping criteria are not met.
*Step 12*: Choose the mode
*Step 13*: If choose Machine Learning Mode
Step 13-1: Sort population and make L-group and H-group
Step 13-2: Learn how to use the L and H groups to create a decision tree.
Step 13-3: Create a new individual *via* hypothesis creation and instantiation
Step 13-4: While termination is not satisfied
*Step 14*: Else (Choose Darwinian Mode)
Step 14-1: Select parents from population according to selection method
Step 14-2: Apply Crossover
Step 14-3: Apply Mutation
Step 14-4: While termination condition
*Step 15*: count increase the number of generations G = G + 1
*Step 16*: Stopping criteria- wait until Gmax reaches 100 or the best objective function hasn’t updated in at least 30 generations before stopping. If all of the stopping criteria are met, proceed to step 18.
*Step 17*: go to step 11
*Step 18*: EXIT
***Output: candidate solution with optimal weight vector for testing phase***

### Evaluation criteria

Since different researches use different functions for testing their methods, selecting suitable evaluation criteria for comparison with other methods is a difficult task because the evaluation criteria employed in different papers are different, and in most cases, the program’s source code is not available. For this reason, in this research, we have tried to use the most common evaluation criteria that have been used in the majority of papers.

The magnitude of Relative Error (MRE) is the most common performance indicator used for measuring the efficiency of software prediction models. MRE calculates the percentage of absolute error against the real effort, as shown in Eq. (5). The mean of MREs, called MMRE, shown in Eq. (6). Also, the median of MREs is called MdMRE.

(5) $$MR{E_i} = \; \displaystyle{{\left| {Estimated\; value - actual\; value} \right|} \over {actual\; value}}$$

(6) $$MMRE = \; \displaystyle{{\mathop \sum_{i = 1}^N MR{E_i}} \over N}$$

PRED(X) is another performance indicator that shows the percentage of predictions correctly predicting *x* value, as defined in Eq. (7).

(7) $$PRED\left( x \right) = \mathop \sum \limits_{i = 1}^N {D_i}*\; \displaystyle{{100} \over N}$$

${D_i} = \left\{ {\matrix{ {1\; \; \; \; \; \; \; \; \; \; \; \; \; \; \; \; \; if\; MMRE < \displaystyle{x \over {100}}} \cr {0\; \; \; \; \; \; \; \; \; \; \; \; \; \; \; \; otherwise\; \; \; \; \; \; \; \; \; \; } \cr } } \right.$

$when\; x = 25\; the\; PRED\; metric\; is\; defind\; as\; PRED\left( {0.25} \right)$

Since most of the papers have used these three criteria for evaluating their proposed methods, we used the same criteria in the current research too. The goal is to minimize MMRE as the final error value and maximize PRED (0.25).

### Training phase

In this phase, a set of training data is given to the model, and the analogy-based cost estimation system is adjusted by features weight, and the LEM algorithm investigates the weight vector in training samples to minimize errors. Except for EFFORT, the rest of the features are considered independent features. Initially, the weight of all features is randomly selected in the range of 0 to 1. Of course, the weights can be equalized, in which case all weights are selected 0 or 1. In this phase, a project is selected in each iteration from the training data, and it is estimated using the proposed equations, *i.e*., the similarity function, *k*, the nearest neighbor, as well as the solution function. The important point in this process is that we will use it to find the optimal weights. Indeed, the weights are suggested by the LEM algorithm and injected into the model. The amount is then estimated as the proposed cost for that project. The proposed cost is compared to the real cost of the project, and its MRE metric is calculated. This process is repeated until all training projects are estimated. In the next step, MMRE and PRED (0.25) are calculated using the MRE values obtained for training data, and these values are passed (sent) to the LEM algorithm. The weights are changed (modified) by the LEM by evaluating the evaluation criteria until the termination criteria (number of repetitions (iterations) or error rate) are met (satisfied) and the weights are recorded as optimized weights. Architecture of this phase has been illustrated in Fig. 2.

### Testing phase

The main purpose of the testing phase is to assess the accuracy of the estimation model made using the testing data. In this phase, the optimized weights obtained from the training phase are applied to the similarity function. Similar to what was done in the training phase, a project is entered into the model and then using the similarity function, K nearest neighbors and solution function, the cost is predicted, and MRE will be counted. This process is repeated for all testing data. Finally, the MMRE and PRED (0.25) evaluation criteria are calculated. The architecture of this phase has been illustrated in Fig. 3.

### Experiment design

In the present research, three popular datasets, *i.e*. Desharnais, Maxwell and ISBSG, were used to evaluate the proposed method. If the accuracy of the cost estimation model is calculated using the projects used to make the model, performance appraisal may be very optimistic, and Estimation error, in this case, maybe significantly lower and may not work well in real-world environments (Hayes, Ryan & Zseller, 1994). In the meantime, the cross-validation approach provides a more realistic, more accurate assessment, in which the data set is divided into several sets of training and testing data. In this paper, 10-fold cross-validation validations are used. In order to design the experiment and to evaluate the proposed method using different experiments, the preprocessing operation has to be run on the data at first. Data preprocessing is one of the most important parts of estimation problems, and its goal is for all features to have the same effect on the target feature. To do so, the data will become normal within the interval [0, 1] using the min-max equation. The normalization operation is done to remove different effects of features (Dejaeger et al., 2011). Then, to train the proposed model, the training datasets have to be divided into training datasets and testing datasets. This was done through the 10-fold cross-validation method in this research. In this division, the datasets are randomly divided into 10 almost-equal sets, among which one set is considered the testing dataset and nine other sets are considered the training datasets.

As mentioned earlier, the analogy-based estimation method has three control parameters, including similarity function, K-nearest neighbor, and solution function. In designing the present experiment, Euclidean and Manhattan distances employed for similarity function and the most common solution functions, *i.e*., median and mean, used to obtain the proposed estimation value. Moreover, the number of closest projects with each other was assumed as variables within the interval of 1 to 5. After specifying the values of parameters, we first defined the effect of different combinations of these parameters in the search space of each parameter and finally selected the most appropriate produced model to be tested with other methods.

## Experiment Results

### Experimenting with the proposed method

To experiment the proposed model, the possibility of obtaining the most desirable configuration of the analogy-based estimation method was investigated. To do so, we used three evaluation criteria, *i.e*., MMRE, PRED (0.25), and MdMRE on Desharnais, Maxwell and ISBSG datasets.

Tables 2–4 show the results of applying the proposed model on the Desharnais and Maxwell datasets, respectively, in which different combinations of the main ABE parameters, which include KNN, similarity function, and solution function, are presented.

**Table 2:** LEMABE results on deshernais dataset.

Similarity	k	Solution		Training set				Testing set		
				MMRE	PRED	MdMRE		MMRE	PRED	MdMRE
Euclidean	K=1	CA		0.25	0.56	0.16		0.34	0.50	0.26
	K=2	mean		0.41	0.47	0.40		0.40	0.48	0.39
		median		0.39	0.43	0.38		0.37	0.46	0.38
	K=3	mean		0.24	0.55	0.17		0.31	0.50	0.25
		median		0.24	0.55	0.17		0.21	0.60	0.15
	K=4	mean		0.28	0.50	0.21		0.27	0.51	0.20
		median		0.24	0.55	0.17		0.26	0.50	0.20
	K=5	mean		0.42	0.21	0.50		0.46	0.21	0.54
		median		0.23	0.57	0.12		0.21	0.61	0.12
Manhattan	K=1	CA		0.36	0.44	0.36		0.35	0.47	0.36
	K=2	mean		0.38	0.36	0.40		0.37	0.32	0.35
		Median		0.48	0.40	0.65		0.46	0.43	0.65
	K=3	mean		0.42	0.25	0.48		0.53	0.20	0.62
		median		0.25	0.54	0.17		0.24	0.55	0.17
	K=4	mean		0.36	0.44	0.30		0.34	0.46	0.28
		median		0.35	0.44	0.30		0.37	0.46	0.32
	K=5	mean		0.27	0.55	0.22		0.28	0.50	0.23
		median		0.41	0.45	0.50		0.49	0.38	0.60

**Table 3:** LEMABE results on maxwell dataset.

Similarity	k	Solution	Training set			Testing set		
			MMRE	PRED	MdMRE	MMRE	PRED	MdMRE
	K=1	CA	0.47	0.46	0.35	0.48	0.46	0.36
Euclidean	K=2	mean	0.66	0.54	0.48	0.56	0.62	0.45
	K=3	mean	0.64	0.46	0.34	0.90	0.38	0.60
		median	0.77	0.58	0.35	0.11	0.61	0.16
	K=4	mean	0.50	0.50	0.30	0.49	0.50	0.30
		median	0.51	0.48	0.29	0.51	0.52	0.29
	K=5	mean	0.66	0.6	0.45	0.56	0.68	0.42
		median	0.67	0.50	0.30	0.62	0.54	0.29
			0.50	0.50	0.30	0.10	0.51	0.12
Manhattan	K=1	median						
	K=2	mean	0.7	0.4	0.47	0.71	0.36	0.49
	K=3	mean	0.62	0.52	0.47	0.53	0.62	0.42
		median	0.64	0.46	0.34	0.90	0.38	0.60
	K=4	mean	0.65	0.62	0.45	0.55	0.63	0.43
		median	0.59	0.52	0.36	0.61	0.44	0.37
	K=5	mean	0.54	0.58	0.33	0.55	0.58	0.33
		median	0.66	0.38	0.34	0.62	0.46	0.31
			0.54	0.6	0.30	0.55	0.68	0.31

**Table 4:** LEMABE results on ISBSG dataset.

Similarity	k	Solution	Training set			Testing set		
			MMRE	PRED	MdMRE	MMRE	PRED	MdMRE
	K=1	CA	0.86	0.29	0.82	0.88	0.3	0.84
Euclidean	K=2	mean	0.71	0.50	0.68	0.79	0.43	0.67
	K=3	mean	0.70	0.50	0.66	0.76	0.39	0.73
		median	0.59	0.40	0.56	0.61	0.59	0.69
	K=4	mean	0.63	0.42	0.60	0.69	0.49	0.64
		median	0.76	0.36	0.70	0.72	0.40	0.75
	K=5	mean	0.67	0.41	0.77	0.77	0.39	0.60
		median	0.59	0.48	0.62	0.58	0.49	0.60
Manhattan	K=1	median	0.82	0.29	0.78	0.85	0.28	0.83
	K=2	mean	0.58	0.55	0.55	0.64	0.52	0.61
	K=3	mean	0.54	0.30	0.55	0.57	0.47	0.59
		median	0.60	0.42	0.59	0.62	0.39	0.60
	K=4	mean	0.50	0.42	0.51	0.50	0.46	0.53
		median	0.51	0.43	0.55	0.70	0.40	0.68
	K=5	mean	0.57	0.40	0.49	0.57	0.44	0.60
		median	0.60	0.38	0.61	0.60	0.38	0.64

**Table 5:** The best model evaluation results related to the three datasets.

Solution function	K	Similarity function	Criterion
Median	3	Euclidean	Best evaluation (considering all three criteria)
Median	5	Euclidean	The lowest value of MdMRE and MMRE
Median	3	Manhattan	The highest value of PRED

According to the proposed framework, evaluating the best option in different criteria was different, but the most suitable and responsive model for all datasets belongs to the Euclidean distance and k-nearest neighbor as well as the median solution function. In addition, the lowest value of MMRE and MdMRE belongs to the Euclidean distance with five nearest neighbors as well as the median solution function, but their PRED value does not contain the highest value among models, although it has relatively suitable values. Based on the obtained results, the highest value of PRED belongs to the Manhattan distance and three nearest neighbors, as well as the median solution function. The summarization from the Best Results of the Model’s Evaluation based on Three Datasets shown in Table 5.

### Comparing the efficiency of LEMABE with other methods

One point to consider while comparing some methods is that the criteria of evaluation functions’ quality and the selected intervals of datasets must be the same so that comparison is made properly. The performance of the proposed framework was validated and compared with the most efficient and commonly used variants of other popular models, namely PSO-based, GA-based feature weight optimization in ABE (GA-ABE), Differential evolution in ABE (DABE), ANN with backpropagation learning-based (de Barcelos Tronto, da Silva & Sant’Anna, 2008; Park & Baek, 2008; López-Martín, 2015), Radial basis function (RBF)-based SDEE (Shin & Goel, 2000). Also, one of the regression-based estimation methods which is multiple regression (MLR) (Mittas & Angelis, 2010), was involved in the comparison process. All of the estimation frameworks using historical data sets and the algorithm parameters were adjusted automatically. The results of such comparisons were applied to different datasets, as shown in Tables 6–8.

**Table 6:** Comparison of LEMABE against other evolutionary algorithms in deshernais dataset.

Methods	MMRE	MdMRE	PRED (0.25)
PSO-based	0.30	0.27	0.54
DABE	0.24	0.23	0.52
ABE	0.42	0.35	0.48
GA-ABE	0.33	0.28	0.57
ANN	0.77	0.69	0.18
RBF	0.53	0.48	0.39
MLR	0.82	0.76	0.22
LEMABE	0.21	0.15	0.60

**Table 7:** Comparison of LEMABE against other evolutionary algorithms in maxwell dataset.

Methods	MMRE	MdMRE	PRED (0.25)
DABE	0.23	0.26	0.53
ABE	0.76	0.78	0.22
RBF	0.46	0.41	0.38
MLR	0.84	0.83	0.29
PSO-based	0.38	0.38	0.47
ANN	0.70	0.72	0.12
GA-ABE	0.19	0.27	0.59
LEMABE	0.11	0.16	0.61

**Table 8:** Comparison of LEMABE against other evolutionary algorithms in ISBSG dataset.

Methods	MMRE	MdMRE	PRED (0.25)
GA-ABE	0.71	0.85	0.45
ANN	0.92	0.94	0.17
ABE	0.88	0.92	0.32
DABE	0.69	0.89	0.39
RBF	0.81	0.83	0.11
PSO-based	0.73	0.81	0.42
MLR	0.98	0.99	0.2
LEMABE	0.61	0.69	0.59

Table 6 shows the results of using the selected estimation methods on the Deshernais dataset based on MMRE, MdMRE and PRED (0.25) criteria. The results show that the proposed model produces more accurate estimates in the testing phase than other methods [MMRE = 0.21, PRED (0.25) = 0.60, MdMRE = 0.15] and subsequently (DABE with values [ MMRE = 0.24, PRED (0.25) = 0.52, MdMRE = 0.23] and the worst estimate was for MLR [MMRE = 0.82, PRED (0.25) = 0.22, MdMRE = 0.76].

The results of applying different estimation models on the Maxwell data set are shown in Table 7. As can be seen in the table, the proposed model has achieved the best performance criteria among other models, the values of which are [MMRE = 0.11, PRED (0.25) = 0.61, MdMRE = 0.16]. GA-ABE was followed by [MMRE = 0.23, PRED (0.25) = 0.53, MdMRE = 0.26]. In addition, MLR produced the worst estimates [MMRE = 0.84, PRED (0.25) = 0.29, MdMRE = 0.83].

Table 8 shows the estimate valuesobtained using the proposed model in the ISBSG dataset. As seen in the table, the performance range of metrics for ISBSG dataset is quite different from the others. The best performance is obtained with [MMRE = 0.61, PRED (0.25) = 0.59, MdMRE = 0.69]. DABE [MMRE = 0.69, PRED (0.25) = 0.58, MdMRE = 0.69] is the closest method to the proposed model and MLR provides the worst estimates for this dataset [MMRE = 0.98, PRED (0.25) = 0.20, MdMRE = 0.99].

**Figure 4:**
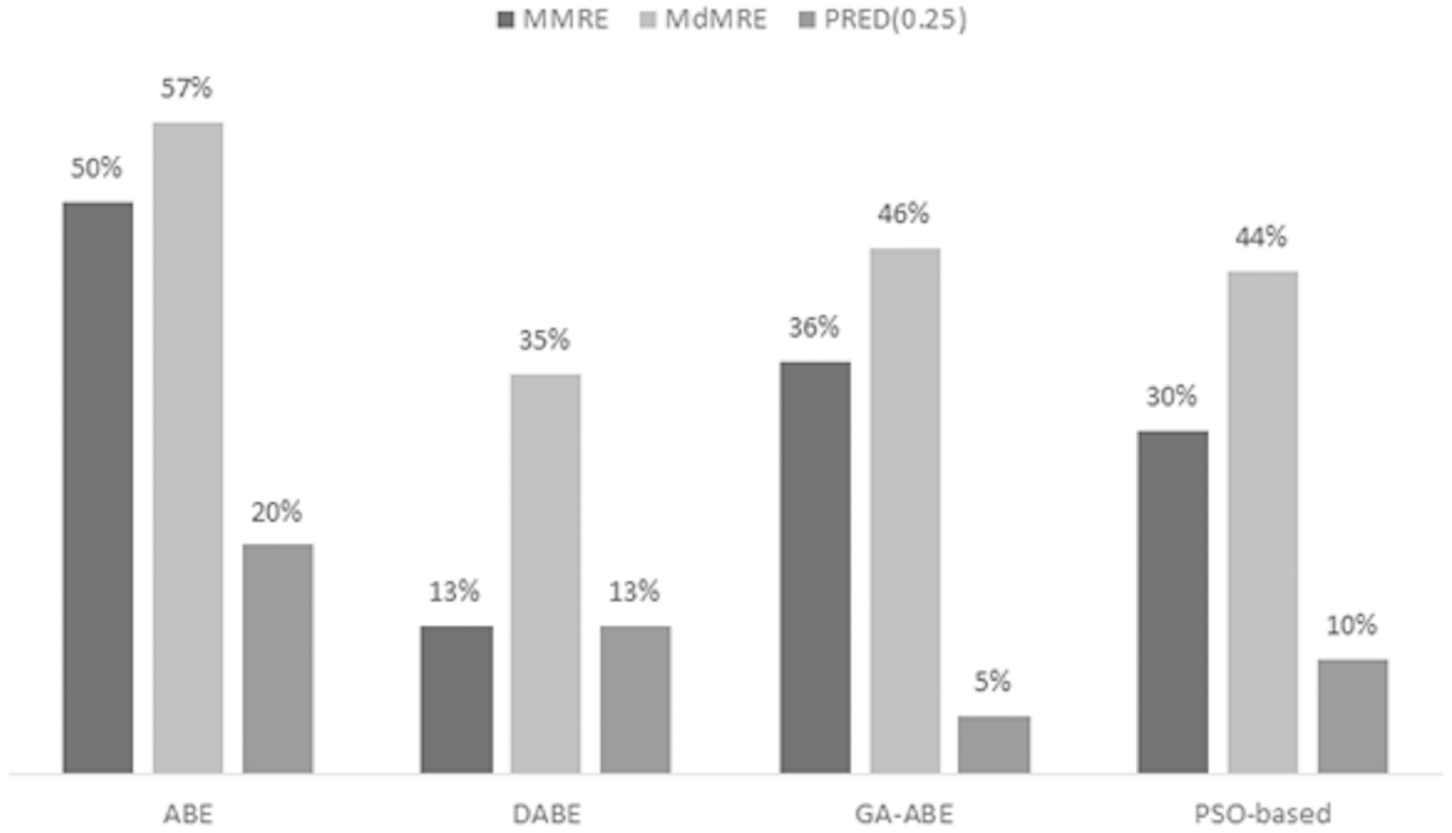
MMRE, MdMRE, and PRED in deshernais dataset in various algorithms.

**Figure 5:**
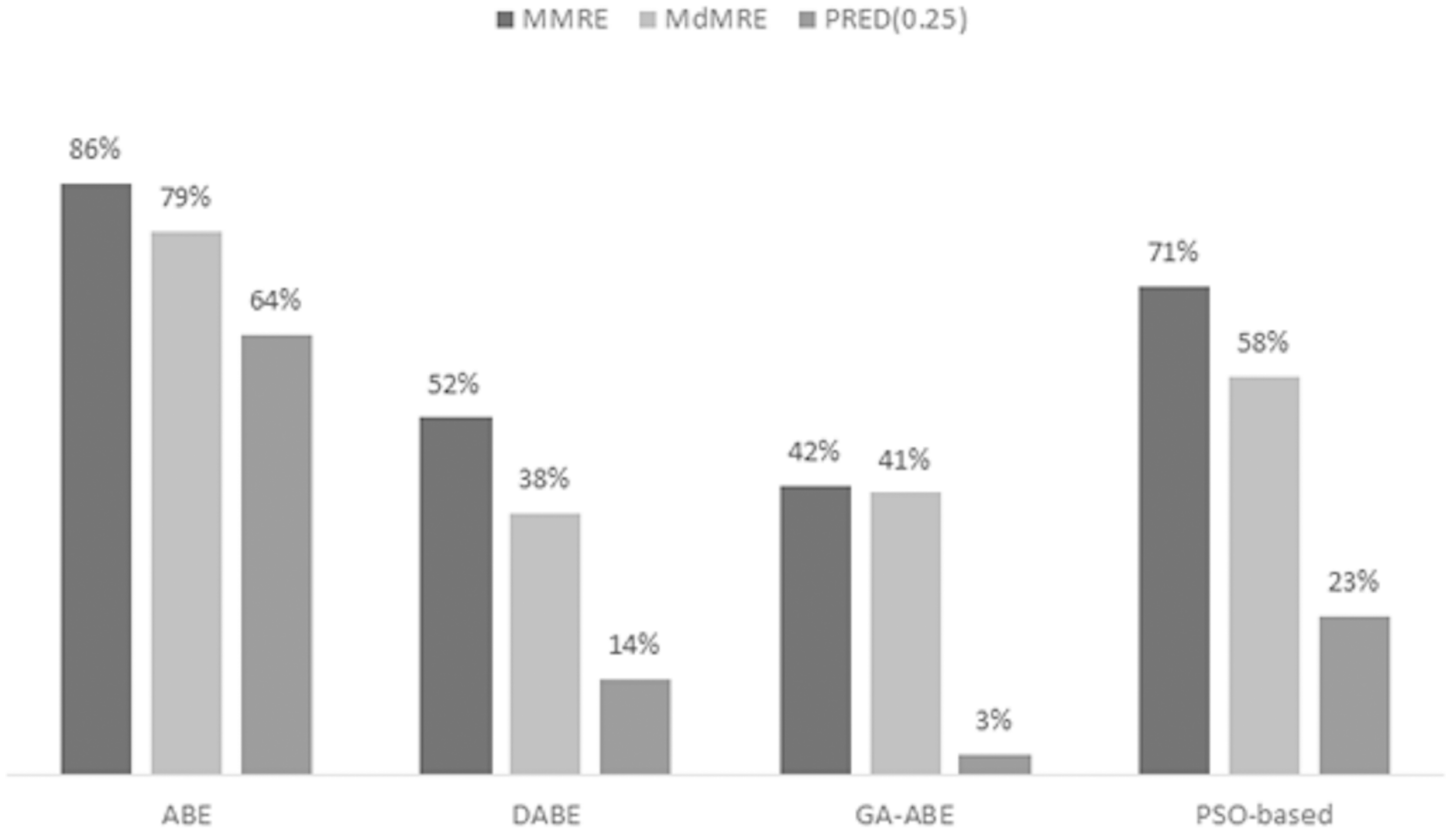
MMRE, MdMRE, and PRED in maxwell dataset in various algorithms.

**Figure 6:**
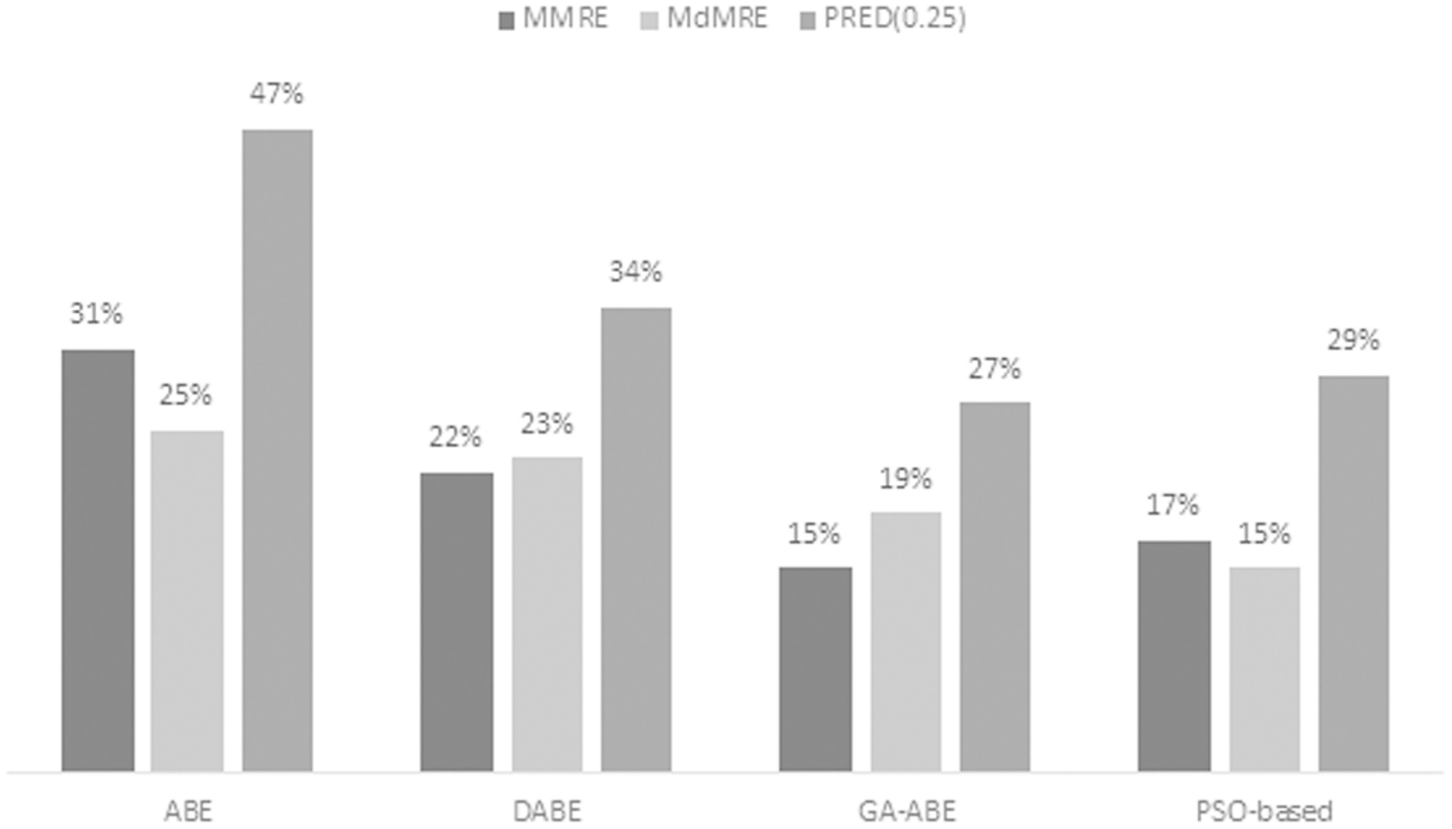
MMRE, MdMRE, and PRED in ISBSG dataset in various algorithms.

As can be seen in the above tables, the value of MMRE and MdMRE criteria is lower than similar values in comparison with other models. Since improving the proposed model and providing comprehensive and clear results is the main goal of this article, to achieve this goal, the percentage improvement of ABE, PSO-based, GA-ABE, DABE models compared to the proposed model are shown in Figs. 4–6. Due to the lack of proper results in ANN, RBF and MLR models, they have been discontinued.

Figure 4 shows the progress made using the proposed model in the Deshernais dataset. According to Fig. 4, the most advanced is the ABE model, in which the evaluation criteria of MMRE, MdMRE, and PRED (0.25) have values of 50%, 57%, and 20%, respectively. The improvement rates of MMRE and MdMRE are almost the same in both PSO-based and GA-ABE models, while in these models, the PRED (0.25) improvement rate is less than 10%. This suggests that the scope of improvement is limited to a few projects. Meanwhile, the percentage of improvement of DABE model has been lower than other models. In general, it can be concluded that in this data set, the progress of MMRE and MdMRE in all estimation models has been higher than PRED (0.25), and LEM can significantly improve the ABE performance in Deshernais data set.

The improvement percentage of the proposed model is shown in Maxwell’s dataset in Fig. 5. It is observed that the proposed model has improved the accuracy of the ABE model in all three criteria of MMRE, MdMRE, and PRED (0.25) by 86%, 79%, and 64%, respectively, which does not indicate the wide range of test projects. A high percentage of accuracy increases that the use of LEM can significantly increase ABE performance in the Maxwell data set. It is also observed that the MMRE and MdMRE criteria for other types of estimation models have also increased significantly. The highest PRED (0.25) progress in this data set occurred with 64%. However, in GA, this value has been minimized, which could be due to the similarity of the internal structure of GA and LEM.

Figure 6 illustrates the percentage of improvement obtained using the proposed model in the ISBSG dataset. It has been observed that the PRED value (0.25) for all types of ABE has increased significantly. Due to the number of outliers and nonnormal projects in ISBSG data set, there has been less improvement in the MMRE and MdMRE criteria.

## Conclusions

One of the most important factors in a successful software project is cost estimation. But because of the Significant growth in size and the variability of requirements, the cost estimation process is difficult and vague. Analogy-based estimation approach is one of the most popular methods of estimating costs, and despite many benefits, ABE is often unable to produce accurate estimates. In the current paper, we proposed a weight optimization technique based on the learnable evolution model in analogy-based estimation. The proposed model was applied to different datasets and tested in different states. The results of the conducted experiments reveal that the results obtained from the proposed model with different evaluation criteria have been satisfactory in most cases. Moreover, the proposed framework was compared with other evolutionary algorithms like genetic algorithm, differential evolution, and particle swarm optimization, according to which more desirable results were obtained. Since the learnable evolution model generates new individuals by a series of production processes and hypotheses, it can to somehow solve the problem of randomness population generation in Darwinian algorithms and increase the speed of convergence as well. This can attract software experts and researchers in the field of cost estimation interested in this algorithm.

## Supplemental Information

10.7717/peerj-cs.800/supp-1 Supplemental Information 1 Implemented code with datasets.

## Additional Information and Declarations

### Data Availability

The following information was supplied regarding data availability: The Desharnais Dataset consisting of Canadian software projects and the Maxwell Dataset consisting of software projects of Finland are available in the Supplemental File.
